# Supplementary figures and images for: Fungal communities represent the majority of root-specific transcripts in the transcriptomes of Agave plants grown in semiarid regions
Source: PeerJ. 2022 May 2;10:e13252. doi: 10.7717/peerj.13252 (PMC9070324; doi:10.7717/peerj.13252)

Total monthly precipitation (mm) in Monteiro, Paraiba, Brazil

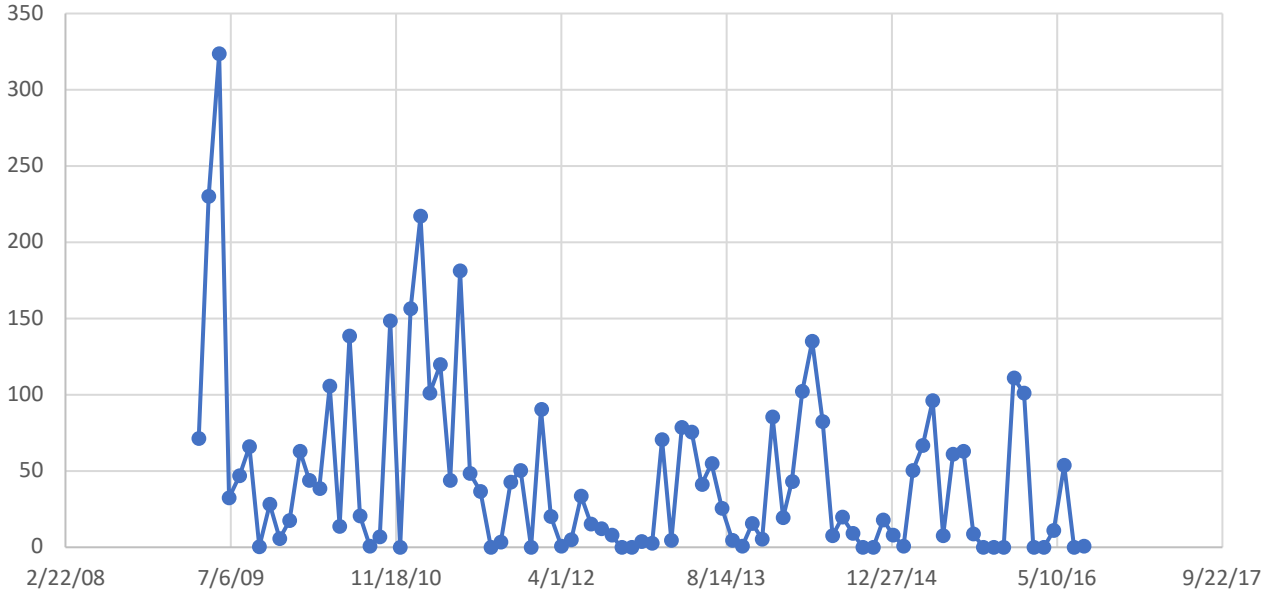

Supplement: Supplemental Information 1 — The germplasm bank where the samples were collected is at a semi-arid climate area with non-calcic brown soil. Before sampling (July 6th, 2016) the precipitation regime was highly irregular. The last precipitation happened on May 31st, 2016. [file peerj-10-13252-s001.pdf]
