# Supplementary material for: Fungal communities represent the majority of root-specific transcripts in the transcriptomes of Agave plants grown in semiarid regions
Source: PeerJ. 2022 May 2;10:e13252. doi: 10.7717/peerj.13252 (PMC9070324; doi:10.7717/peerj.13252)
Supplement: Supplemental Information 2 — To obtain all heat shock proteins in our fungal datasets and the fungal genomes (1,014 genomes), we searched with HMMER (E-value threshold of 0.001). We used the distribution pattern to select the Ascomycota and Basidiomycota genomes with more HSPs, which accounted for the top 10 genomes each. [file peerj-10-13252-s002.pdf]

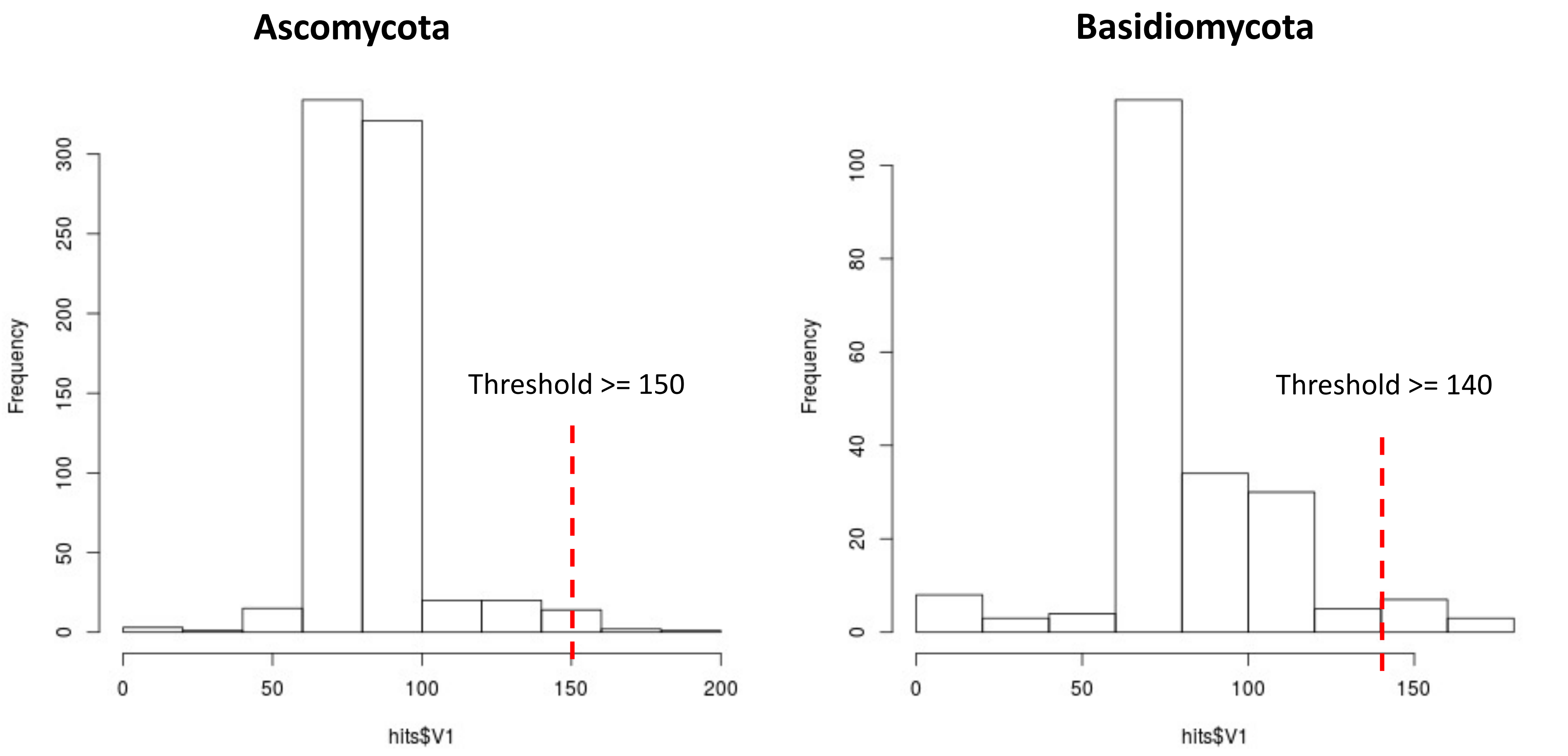

Distribution of HSP family members in the asco (left) and basidio (right) genomes
