# Supplementary material for: Fungal communities represent the majority of root-specific transcripts in the transcriptomes of Agave plants grown in semiarid regions
Source: PeerJ. 2022 May 2;10:e13252. doi: 10.7717/peerj.13252 (PMC9070324; doi:10.7717/peerj.13252)
Supplement: Supplemental Information 12 — We used TPR and FPR values to compare parameters for classification of Ascomycota and Basidiomycota (see Methods section). The script is also available as a Supplemental File. [file peerj-10-13252-s012.pdf]

S7 - Supplemental Results for taxonomic analysis pipeline

The comparison based on amino acid similarity (tBLASTx), in function of top N BLAST hits (N from 1 to 4), greatly reduces the number of unclassified sequences compared to BLASTn (Fig. 1A). Regarding the classification between the Ascomycota and Basidiomycota phyla, the metric TPR (Fig. 1B) shows that Ascomycota is better classified than Basidiomycota and both have low FPR (below 0,5% for N >= 2; Fig. 1C). Thus, we consider the E-value <= 1e-10 and N>=2 as the threshold for the next analyses.

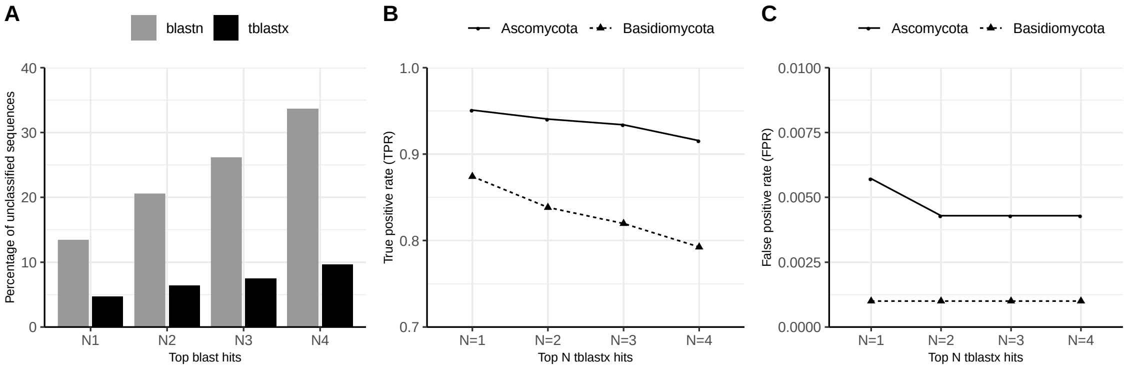

Figure 1: Testing of different parameters in the pipeline. A: Comparison between using BLASTn or tBLASTx to decrease the number of unclassified sequences, both using the same E-value threshold. Note that tBLASTx presented a lower percentage of unclassified. B: True positive rate (TPR) comparison between the number of top N tBLASTx hits. C: False positive rate (FPR) comparison between the number of top N tBLASTx hits.

After parameter optimization step, we applied the pipeline to classify the fungi transcripts of *A. sisalana*, hybrid 11648 and *A. fourcroydes* among the Ascomycota, Basidiomycota, Asco or Basidio, and Other Fungi groups (Table 2 on manuscript).
